# Variation in human gut microbiota impacts tamoxifen pharmacokinetics

**DOI:** 10.1128/mbio.01679-24

**Published:** 2024-11-25

**Authors:** Yasmine Alam, Sheron Hakopian, Lizett Ortiz de Ora, Ian Tamburini, Julio Avelar-Barragan, Sunhee Jung, Zane Long, Alina Chao, Katrine Whiteson, Cholsoon Jang, Elizabeth Bess

**Affiliations:** 1 Department of Biological Chemistry, University of California Irvine, Irvine, California, USA; 2 Department of Pharmaceutical Sciences, University of California Irvine, Irvine, California, USA; 3 Department of Chemistry, University of California Irvine, Irvine, California, USA; 4 Department of Molecular Biology & Biochemistry, University of California Irvine, Irvine, California, USA; 5 Chao Family Comprehensive Cancer Center, University of California Irvine, Irvine, California, USA; 6 Center for Complex Biological Systems, University of California Irvine, Irvine, California, USA; 7 Center for Epigenetics and Metabolism, University of California Irvine, Irvine, California, USA; University of Maryland School of Medicine, Baltimore, Maryland, USA

**Keywords:** human gut microbiome, tamoxifen, breast cancer

## Abstract

Tamoxifen is the most prescribed drug used to prevent breast cancer recurrence, but patients show variable responses to tamoxifen. Such differential inter-individual response has a significant socioeconomic impact as one in eight women will develop breast cancer and nearly half a million people in the United States are treated with tamoxifen annually. Tamoxifen is orally delivered and must be activated by metabolizing enzymes in the liver; however, clinical studies show that neither genotype nor hepatic metabolic enzymes are sufficient to predict why some patients have sub-therapeutic levels of the drug. Here, using gnotobiotic- and antibiotics-treated mice, we show that tamoxifen pharmacokinetics are heavily influenced by gut bacteria and prolonged exposure to tamoxifen. Interestingly, 16S rRNA gene sequencing shows tamoxifen does not affect overall microbiota composition and abundance. Metabolomics, however, reveals differential metabolic profiles across the microbiomes of different donors cultured with tamoxifen, suggesting an enzymatic diversity within the gut microbiome that influences response to tamoxifen. Consistent with this notion, we found that β-glucuronidase (GUS) enzymes vary in their hydrolysis activity of glucuronidated tamoxifen metabolites across the gut microbiomes of people. Together, these findings highlight the importance of the gut microbiome in tamoxifen’s pharmacokinetics.

**IMPORTANCE:**

One in eight women will develop breast cancer in their lifetime, and tamoxifen is used to suppress breast cancer recurrence, but nearly 50% of patients are not effectively treated with this drug. Given that tamoxifen is orally administered and, thus, reaches the intestine, this variable patient response to the drug is likely related to the gut microbiota composed of trillions of bacteria, which are remarkably different among individuals. This study aims to understand the impact of the gut microbiome on tamoxifen absorption, metabolism, and recycling. The significance of our research is in defining the role that gut microbes play in tamoxifen pharmacokinetics, thus paving the way for more tailored and effective therapeutic interventions in the prevention of breast cancer recurrence.

## INTRODUCTION

The societal impact of breast cancer is substantial: one in eight women will develop breast cancer in their lifetime and nearly half a million people with this disease in the United States are treated with tamoxifen each year (1–4). Tamoxifen is the most commonly prescribed orally administered estrogen receptor antagonist used to prevent hormone-dependent breast cancer recurrence (1–3). However, there is a high degree of variability in interindividual responses (3). Over the 5- to 10-year course of tamoxifen treatment, the drug’s therapeutic effects can be dampened, leading to one in three people experiencing breast cancer recurrence (5). The underlying mechanisms of such interindividual variations are poorly understood. The long-standing dogma is that variable drug response is attributed to inter-patient variations in the expression of drug-metabolizing enzymes and efflux proteins (5). However, this mechanism predicts only a small portion of the variability (6–8). These observations strongly suggest the existence of additional key factors modulating tamoxifen efficacy. One potential factor can be differential tamoxifen pharmacokinetics (i.e., variable absorption and clearance of the drug from the bloodstream).

Tamoxifen is a prodrug that undergoes a process of bioactivation in the liver, whereby it is transformed through 4-hydroxylation and *N*-demethylation, leading to the production of (*Z*)-4-hydroxytamoxifen and (*Z*)-endoxifen, respectively. Both 4-hydroxytamoxifen and endoxifen demonstrate significantly greater anti-estrogenic potency, 30–100 times stronger, than tamoxifen (9, 10). Although conversion to 4-hydroxytamoxifen accounts for only 7% of tamoxifen metabolism, endoxifen’s plasma concentrations in individuals undergoing therapy with tamoxifen are typically at least 10 times higher (6, 10). Specifically, a therapeutic endoxifen threshold of >16 nM, first reported by Madlensky et al., was inversely associated with the risk of breast cancer recurrence (11). The study showed a 26% lower disease-free survival rate in patients with endoxifen levels <16 nM as compared to those with >16 nM endoxifen levels. Of note, at the current standard dose of tamoxifen, 20 mg once daily, approximately 20–24% of patients have endoxifen levels less than 16 nM (6, 11).

**Fig 1:**
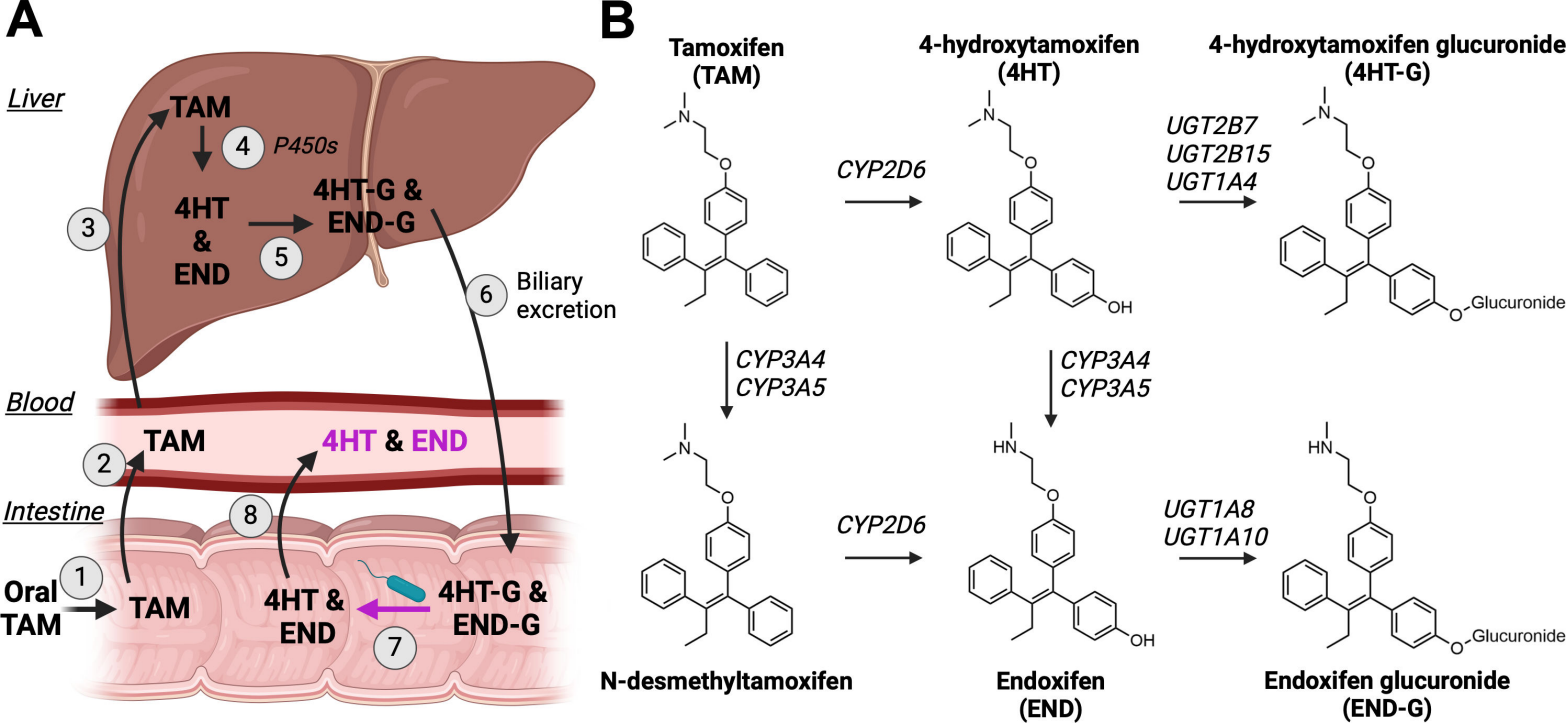
Tamoxifen metabolism and enterohepatic circulation. (**A**) (1) Oral administration of TAM results in the exposure of the gut microbiota to the drug. (2) After absorption into the portal circulation and (3) transport to the liver, (4) tamoxifen undergoes bioactivation by P450 enzymes to produce endoxifen (END) and 4-hydroxytamoxifen (4HT) through two pathways: 4-hydroxylation and *N*-demethylation. (5) These metabolites undergo glucuronidation, leading to the formation of endoxifen glucuronide (END-G) and 4-hydroxytamoxifen glucuronide (4HT-G), respectively. (6) Approximately 75% of END-G and 4HT-G are excreted in the bile to the intestine. (7) END-G and 4HT-G necessitate hydrolysis by β-glucuronidase (GUS) (8) before END and 4HT can be reabsorbed into the systemic circulation and exert anti-cancer effects. (**B**) Chemical structures of tamoxifen and key metabolites and the liver enzymes responsible for their production.

Levels of endoxifen in the bloodstream substantially differ across individuals undergoing treatment with tamoxifen. A contributing factor to these interindividual differences is the variable activity of hepatic cytochrome P450 enzymes involved in endoxifen production. Specifically, tamoxifen is metabolized to endoxifen by CYP3A4 or CYP3A5 that performs *N*-demethylation, and CYP2D6 which performs hydroxylation (Fig. 1) (12). Despite the critical role of cytochrome P450s in tamoxifen’s bioactivation, genetic variations in these enzymes are not adequate predictors of the variable serum endoxifen levels observed across people (5). Moreover, only 46% of the variance in endoxifen concentrations could be attributed to other factors (i.e., age, BMI, genotype), suggesting that there are other factors that play a role in variable tamoxifen metabolite concentrations (11).

Bacteria in the gastrointestinal (GI) tract—the gut microbiota—are collectively recognized as a vital “metabolic organ” that can influence the accessibility and effectiveness of orally administered medicinal compounds. Among these, tamoxifen serves as a prime example of the putatively intricate interplay between the gut microbiome and drug efficacy, given its repeated interaction with gut microbes. However, there is still much to be discovered about the microbial factors impacting drug disposition, that is, pharmacomicrobiomics (13, 14). Even before metabolism begins in the liver, microbial enzymes can metabolize drugs and chemically transform them into bioactive, inactive, or potentially toxic metabolites (15). This, in turn, alters the bioavailability of ingested compounds, thus impacting the efficacy of therapeutic drugs (6).

Humans harbor extensive and uniquely varied microbial communities, with the GI tract being the most abundant reservoir (16). Approximately 75% of tamoxifen is eliminated through bile in the gastrointestinal (GI) tract (15, 17), leading to a notable concentration of this drug in the GI environment, estimated at 20 µM in the small intestine (15, 18). We thus hypothesize that the variable effectiveness of tamoxifen across people is likely influenced by the gut microbiota (19). Figure 1 illustrates the chemical conversion of tamoxifen *in vivo*, highlighting putative key steps in the metabolism of this drug. In the liver, drugs are typically subjected to various metabolic pathways to transform lipid-soluble bioactive compounds into water-soluble inactive forms, facilitating their elimination from the body through the kidneys and bile. One such mechanism is the glucuronidation of compounds, which is the primary means of liver metabolism for tamoxifen and its metabolites (20). Because the gut microbiome encodes β-glucuronidase (GUS) enzymes, capable of hydrolyzing glucuronide intermediates, we sought to investigate how the interplay between tamoxifen and gut microbiome GUS enzymes can impact drug metabolism and disposition.

GUS enzymes, which vary in abundance across the gut microbiomes of people, may be a missing link to the effective prediction of serum endoxifen levels. In the liver, endoxifen is glucuronidated by enzymes in the diverse family of UDP-glucuronosyltransferases (21, 22) and is subsequently directed to the intestine through biliary excretion. However, as much as 69% of the metabolites in the bile are reabsorbed by enterohepatic recirculation (15, 23). Returning endoxifen to systemic circulation where it can exert its anti-cancer effects depends on hydrolysis of endoxifen–glucuronide; we sought to determine whether this hydrolysis could be performed by gut bacterial GUS enzymes.

Although GUS activity is abundant in the gut microbiome, there are six unique structural categories into which 279 distinct microbiome-encoded GUS proteins are classified (24). The varied substrate scope of these enzymes combined with their varied abundance across human gut microbiomes results in differing abilities to hydrolyze glucuronidated metabolites. This variability in GUS activity across people may explain the variations in serum endoxifen levels. Here we report data from mouse experiments, *ex vivo* incubations of human fecal samples, enzyme extract activity, and bacterial metagenomic analyses of fecal samples to uncover variations in gut bacterial GUS activity that can impact tamoxifen’s therapeutic efficacy.

## RESULTS

### Variation in tamoxifen pharmacokinetics as a function of the gut microbiome

**Fig 2:**
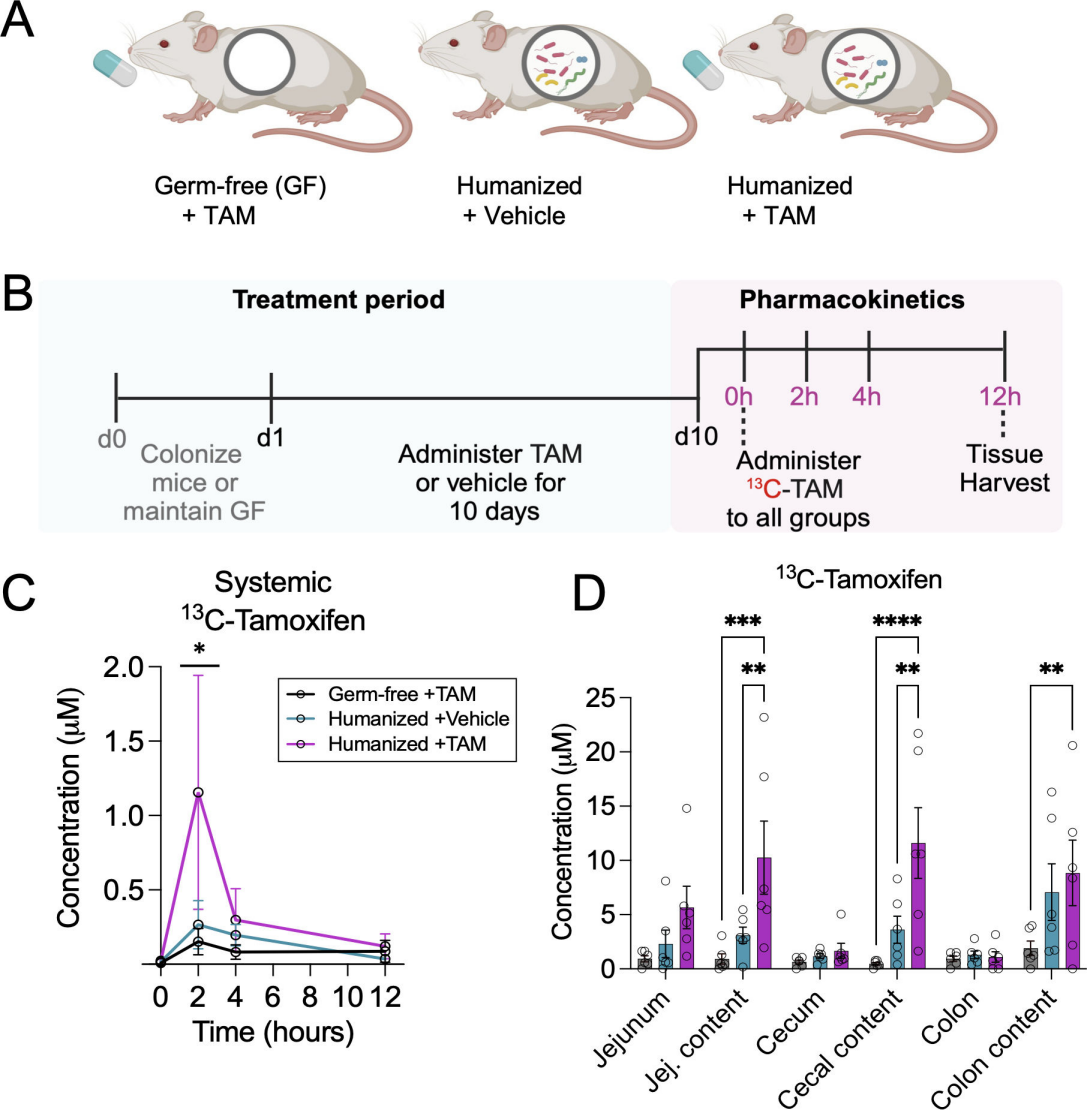
Tamoxifen pharmacokinetics as a function of the gut microbiota. (**A**) Experimental groups (*n* = 6/group). (**B**) Experimental timeline. Germ-free mice were colonized with human fecal bacteria and tamoxifen was administered daily for 10 days. On day 10, all mice were dosed with 1:1 uniformly labeled ^13^C tamoxifen:natural-isotope-abundance tamoxifen to profile pharmacokinetics over a 12-hour time course. (**C**) Circulating levels of ^13^C-tamoxifen across groups. (**D**) ^13^C-tamoxifen abundance across groups in intestinal tissue and luminal contents. Data are mean ± standard error (s.e.). *P < 0.05, **P < 0.01, ****P < 0.0001 by a two-way ANOVA and Tukey’s multiple comparisons test.

Gnotobiotic mouse models remain an excellent model for understanding the role of gut microbiota in health and disease. To assess whether bacterial species in complex communities can interact with tamoxifen and alter its bioavailability, germ-free (GF)-BALB/c mice were either maintained GF or colonized with a cocktail of fecal samples from five healthy human donors (collected under anaerobic conditions according to our IRB-approved protocol). All mice were maintained in a gnotobiotic isolator, and we routinely confirmed the sterility of the GF mice by aerobic and anaerobic culturing and PCR of freshly passed fecal pellets. Following a 1-week adjustment period to the humanization with donor communities, mice were then treated daily with tamoxifen (20 mg/kg) (25, 26) or vehicle (corn oil) for 10 days via oral gavage to mimic long-term tamoxifen treatment in patients and explore whether prolonged exposure to tamoxifen may alter gut microbial response (17) (Fig. 2A and B). Gavages were administered in the evening just before the mice began eating to replicate the timing that patients typically consume the drug, minimizing the potential effect of circadian factors (27, 28). The daily gavages did not affect body weight or normal activity. After 10 days, all mice were dosed with a 1:1 mixture of ^13^C-tamoxifen and unlabeled tamoxifen (20 mg/kg), and blood was collected at multiple time points to determine tamoxifen blood pharmacokinetics and drug–microbe interactions. Liquid chromatography–mass spectrometry (LC–MS) was then used to quantitate tamoxifen levels in blood and tissues. Tamoxifen level in blood was significantly enhanced in humanized mice treated daily with tamoxifen, compared to GF mice treated with tamoxifen or humanized mice treated with vehicle alone (Fig. 2C).

At the terminal endpoint, we harvested relevant gastrointestinal organs. In the small intestine, only jejunal contents were collected because the passage of drugs through the duodenum is relatively rapid with low absorption, and the ileum has fewer villi than the jejunum and thus also has lower net absorption (29). Prolonged exposure to tamoxifen markedly increased ^13^C-tamoxifen accumulation in both small and large intestinal contents (jejunum, cecum, and colon) (Fig. 2D). This was observed 12 hours after the drug administration when the drug was already cleared from circulation (Fig. 2C). Intriguingly, compared to GF mice, humanized mice treated with vehicle alone showed slightly higher tamoxifen levels, though not statistically significant. Thus, prolonged exposure to the drug combined with the presence of bacteria led to the highest levels of circulating tamoxifen. This suggests that the gut microbiome may have adapted to tamoxifen, whether compositionally or functionally at the enzymatic level, and may be capable of regulating the reabsorption of its bioactive metabolites into the bloodstream.

**Fig 3:**
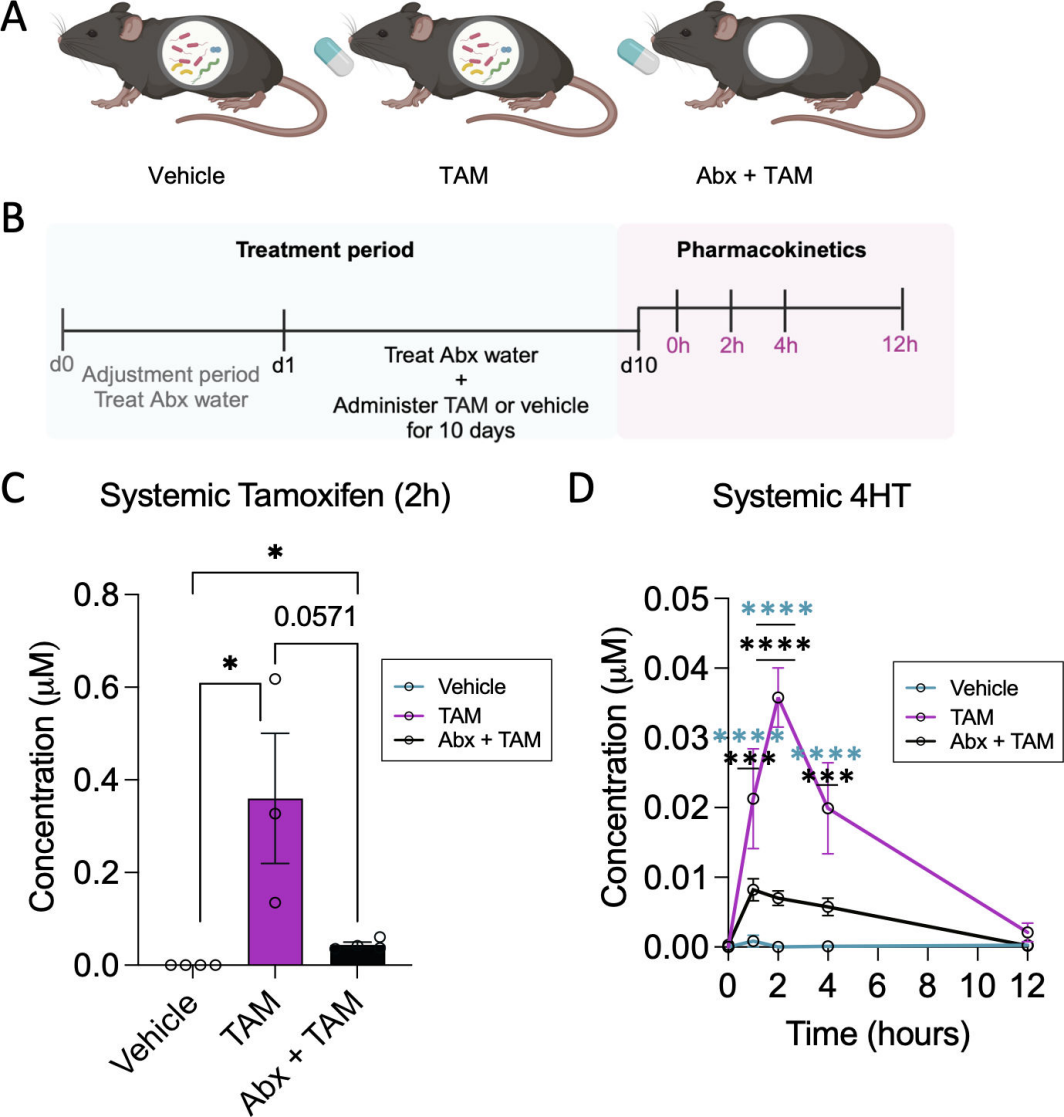
Tamoxifen pharmacokinetics in antibiotics-treated mice. (**A**) Experimental groups *n* = 4, 3, 4 per group (Vehicle, TAM, Abx + TAM). (**B**) Experimental timeline. Antibiotics and tamoxifen or vehicle were administered daily for 10 days. All mice were dosed with tamoxifen to profile pharmacokinetics over a 12-hour time course. (**C and D**) Circulating levels of tamoxifen at the 2-hour peak time point (**C**) and 4HT across time points (**D**). Data are mean ± s.e. *P < 0.05, ***P < 0.001, ****P < 0.0001 by unpaired nonparametric Kolmogorov–Smirnov t-tests and two-way ANOVA with Tukey’s multiple comparisons test.

Next, we recapitulated these findings in a standard specific-pathogen-free C57BL/6J mouse model using a cocktail of antibiotics (ampicillin, neomycin, metronidazole, and vancomycin) to deplete the numbers of gut bacteria. Following a 4-day adjustment period to the antibiotics, mice were then treated daily with tamoxifen (20 mg/kg) or vehicle for 10 days via oral gavage and maintained on their antibiotics regimen (Fig. 3A and B). After 10 days, all mice were dosed with tamoxifen or vehicle, and blood was collected at multiple time points to determine tamoxifen blood pharmacokinetics and drug–microbe interactions. Consistent with the gnotobiotic study, antibiotics treatment trended (*P* = 0.0571) to lower tamoxifen peak blood levels (Fig. 3C). Furthermore, antibiotics substantially reduced 4HT levels in blood, an important finding given that 4HT is an active metabolite of tamoxifen (Fig. 3D). Intriguingly, tamoxifen had no effect on bacterial load as measured by qPCR of the 16S rRNA gene (Fig. S1). Together, these *in vivo* data suggest that gut microbiota impact tamoxifen pharmacokinetics and, therefore, likely affect therapeutic efficacy.

**Fig 4:**
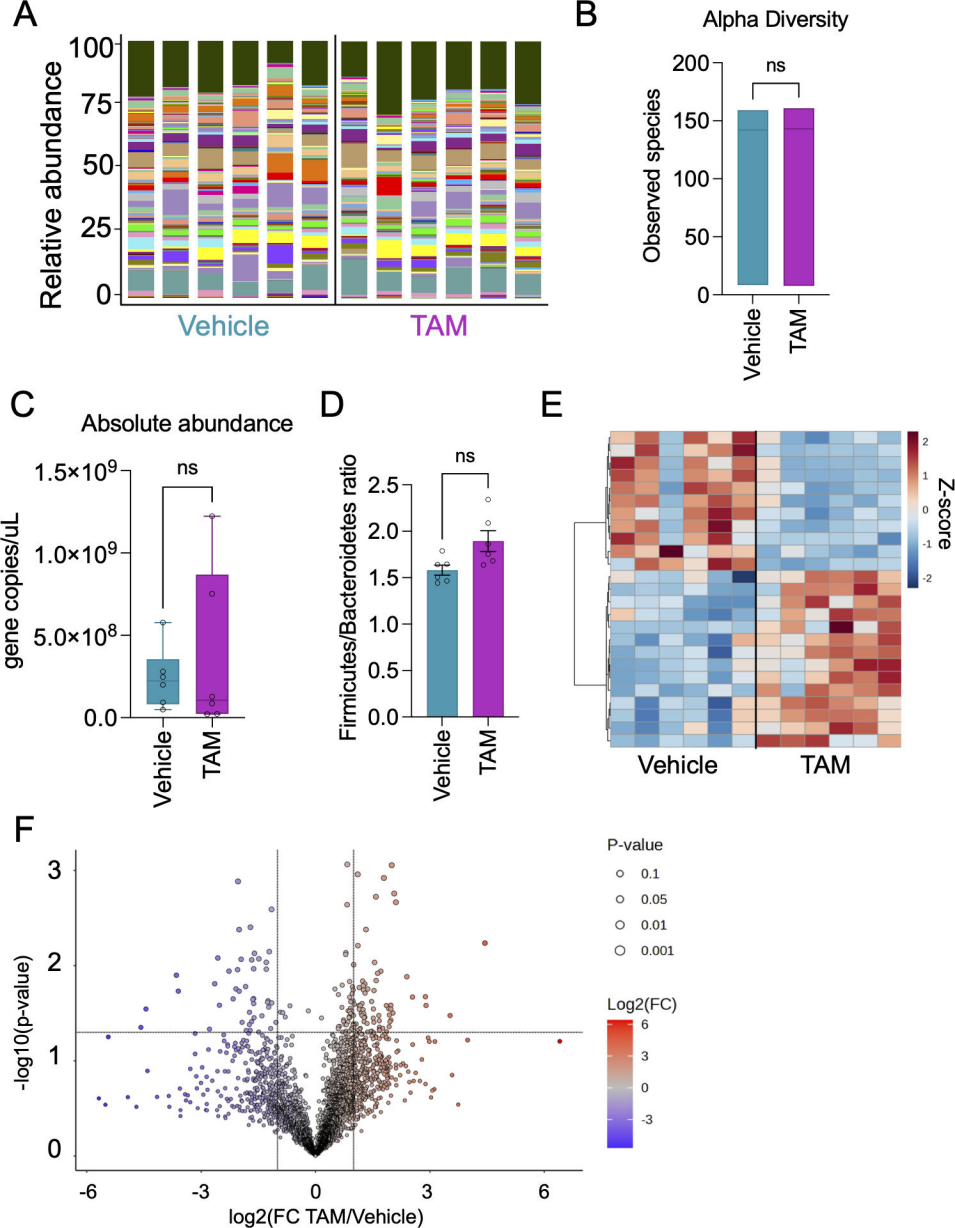
Diversity, abundance, and metabolome of bacteria in the gut microbiome of tamoxifen-treated mice. (**A**) Taxonomic analysis of microbial composition at the species level from cecal samples of the humanized + TAM and the humanized + vehicle mouse groups. The size of the bars represents the relative abundance of the bacterial taxa. Different species are shaded by different colors; see raw data (Table S1) for legend. Tamoxifen-treated mice showed no difference in (**B**) alpha diversity (richness) or (**C**) abundances as measured by copies of the 16S rRNA gene compared to vehicle. (**D**) Firmicutes/Bacteroidetes ratio of all samples. (**E**) Heatmap of the top 25 significant metabolites identified in the cecal content of humanized mice. Rows indicate metabolites (Table S4) and columns represent each mouse sample. (**F**) Volcano plot broadly indicates the statistically significant metabolites between groups at a significance threshold of 0.05. FC: fold change. *N* = 6 mice/group. Data are mean ± s.e. Unpaired nonparametric Kolmogorov–Smirnov t-tests were used to calculate and compare cumulative distributions between groups.

Our observation that prolonged tamoxifen treatment increases blood tamoxifen concentration when in the presence of human gut bacteria (Fig. 2C) motivated us to determine whether tamoxifen treatment changes gut microbiota composition. We thus performed 16S rRNA gene amplicon analysis of bacteria in gnotobiotic mouse cecums. Surprisingly, we observed almost no change in the relative abundances of bacterial species (Fig. 4A; Table S1). The most abundant phyla in tamoxifen-treated mice, on average, were *Firmicutes* accounting for 46.7%, *Bacteroidetes* for 29.6%, and *Verrucomicrobia* for 21%, while vehicle-treated mice were 52.4%, 27.8%, and 17.1%, respectively (Fig. S2A; Table S2). At the genus level, *Akkermansia* dominated (21%) in tamoxifen-treated mice, followed by *Bacteroides* (16.8%), *Blautia* (10.5%), and *Parabacteroides* (7.9%) (Fig. S2B; Table S3). Moreover, there was no difference in microbial alpha diversity (richness) or total abundance between vehicle or tamoxifen-treated groups (Fig. 4B and C). The Firmicutes to Bacteroidetes ratio also showed no significant difference between groups (Fig. 4D). Therefore, overall microbial composition and abundance are not sufficient to explain differences in tamoxifen pharmacokinetics.

Furthermore, we performed metabolomic analysis on the cecal contents from the humanized mice in the gnotobiotic study (Fig. 4E and F; Table S4). Broadly, there were several putative metabolites that differed between treatment groups, though all were unannotated upon referencing our in-house library and other online databases. Of the more than 5,800 putative metabolites that were detected, 117 were significantly higher in the humanized + TAM group while 75 were significantly enriched in the humanized + vehicle group (Fig. 4F).

To determine whether the abundance of any bacterial taxa correlates with circulating levels of ^13^C-tamoxifen (as measured by the area under the curve in Fig. 2C), we performed a correlation analysis. No significant correlation between bacterial taxa and ^13^C-tamoxifen AUC was detected at a significance threshold of *P* < 0.05 (FDR correction) (Table S5). However, comparing ^13^C-tamoxifen peak levels at 2 hours post-administration with bacterial taxa abundance yielded two significant correlates: Clostridia (Firmicutes; Spearman correlation = 0.855, FDR-corrected *P*-value = 0.0466) and Erysipelotrichia (Firmicutes; Spearman correlation = 0.854, FDR-corrected *P*-value = 0.0466) (Table S6). Of note, Clostridia express GUS (30), and the abundance of each of the identified taxa is positively linked to GUS activity (31, 32).

### Inter-individual variation in human gut microbiota response to tamoxifen

**Fig 5:**
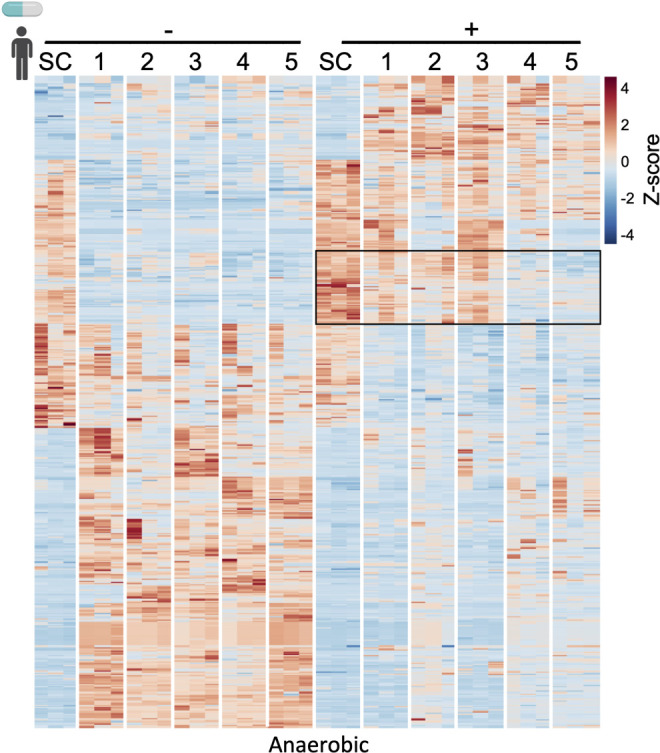
Metabolomic analysis of tamoxifen-treated human gut bacterial cultures. (**A**) Heatmap showing relative abundances of known and unknown metabolites in *ex vivo* cultures of fecal samples incubated with tamoxifen (right) or vehicle control (left). Rows indicate metabolites (Table S7) and columns represent each donor sample. *N* = 5 fecal samples (one per donor) and three biological replicates per sample. SC, sterile control (without bacteria). Boxed region indicates metabolites that are enriched in donors 1–3 only. Only anaerobic condition is shown here; data for aerobic condition are shown in Fig. S3; Table S8.

We next sought to determine how tamoxifen changes the metabolic landscape of the human gut microbiota. Five healthy human donors’ fecal samples were cultured with a physiologically relevant dose of tamoxifen (60 µM), the predicted concentration of tamoxifen in the colon (18), or vehicle alone for 72 hours, in either anaerobic or aerobic conditions to capture the broad range of bacteria that can grow within the oxygen gradient that exists in the human intestinal tract. Then, targeted and untargeted metabolomics were performed on these samples. The data revealed overall similar metabolomic profiles between the five donors in the absence of tamoxifen (Fig. 5; Fig. S3; Tables S7 and S8). By contrast, in the presence of tamoxifen, some variability in metabolomic profiles emerged across donors. For example, many putative metabolites were enriched in the presence of tamoxifen in donors 1–3 but not in donors 4 or 5 (Fig. 5). Intriguingly, of the metabolites that increased in abundance following tamoxifen exposure in either aerobic or anaerobic conditions, most were fatty acids (Tables S7 and S8). Altogether, these tamoxifen-induced shifts in gut bacterial metabolomic profiles point to inter-individual variations in gut microbiome–tamoxifen interactions.

### Levels of tamoxifen and associated metabolites as a function of glucuronidase activity

**Fig 6:**
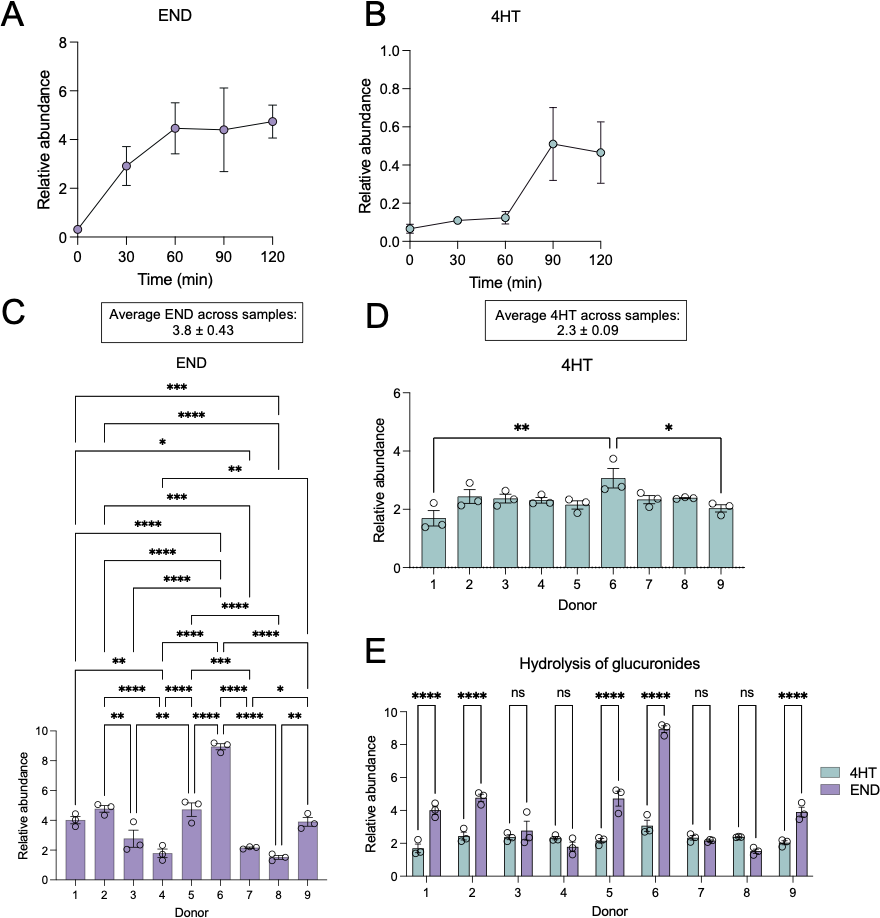
Distinct GUS activities across gut microbiomes from different healthy human donors. Time-course profile following incubation of fecal lysate from donor 6, the sample that is the highest producer of END, with (**A**) END-G or (**B**) 4HT-G for 120 minutes. Levels of hydrolyzed (**C**) END or (**D**) 4HT following incubation of lysates from 9 different fecal samples with glucuronidated 4-HT and END for 180 minutes. Average across donors ± s.e. boxed above graphs for each hydrolyzed product. (**E**) Comparison of hydrolysis of 4HT-G and END-G per donor. *N* = 3 technical replicates per donor. Data are mean ± s.e. *P < 0.05, **P < 0.01, ***P < 0.001, ****P < 0.0001 by ordinary one-way ANOVA and Tukey’s multiple comparisons test. Comparisons not shown were not statistically significant. ns, not significant.

Because 75% of tamoxifen and its bioactive metabolites are glucuronidated (15) and, upon biliary excretion, are subsequently exposed to intestinal bacteria that can express glucuronide-hydrolyzing GUS enzymes, we hypothesized that this metabolite–bacteria interaction shapes tamoxifen metabolism. We examined this relationship by incubating 4HT-G and END-G with protein in crude lysates of fecal samples from nine people. After 180 minutes at 37°C, glucuronide hydrolysis was quantified by measuring 4HT and END concentrations using LC–MS. Cleavage of END-G to END occurs relatively quickly, beginning before 30 minutes post-incubation with human fecal microbiota (Fig. 6A). By contrast, bacterial enzymes responsible for cleaving 4HT-G require more time, with 4HT abundance peaking approximately 90 minutes post-incubation with gut bacteria (Fig. 6B). Hydrolysis of END-G was highly variable across fecal samples, although there was little variation for 4HT-G hydrolysis (Fig. 6C and D). For example, donors 4 and 8 demonstrated little hydrolysis of END-G, while donors 1, 2, 5, and 9 were moderate hydrolyzers, with donor 6 exerting the most hydrolysis activity. Comparing hydrolysis of 4HT-G and END-G per donor shows that donors 1, 2, 5, 6, and 9 likely have greater enzymatic efficiency for the hydrolysis of END-G than the other four donors (Fig. 6E). The variation in END-G hydrolysis combined with the similarity in 4HT-G metabolism across fecal samples suggests that the enzymes responsible for hydrolysis have different specificities for these two substrates.

#### Correlation analysis of fecal bacteria GUS gene abundance to GUS activity

**Fig 7:**
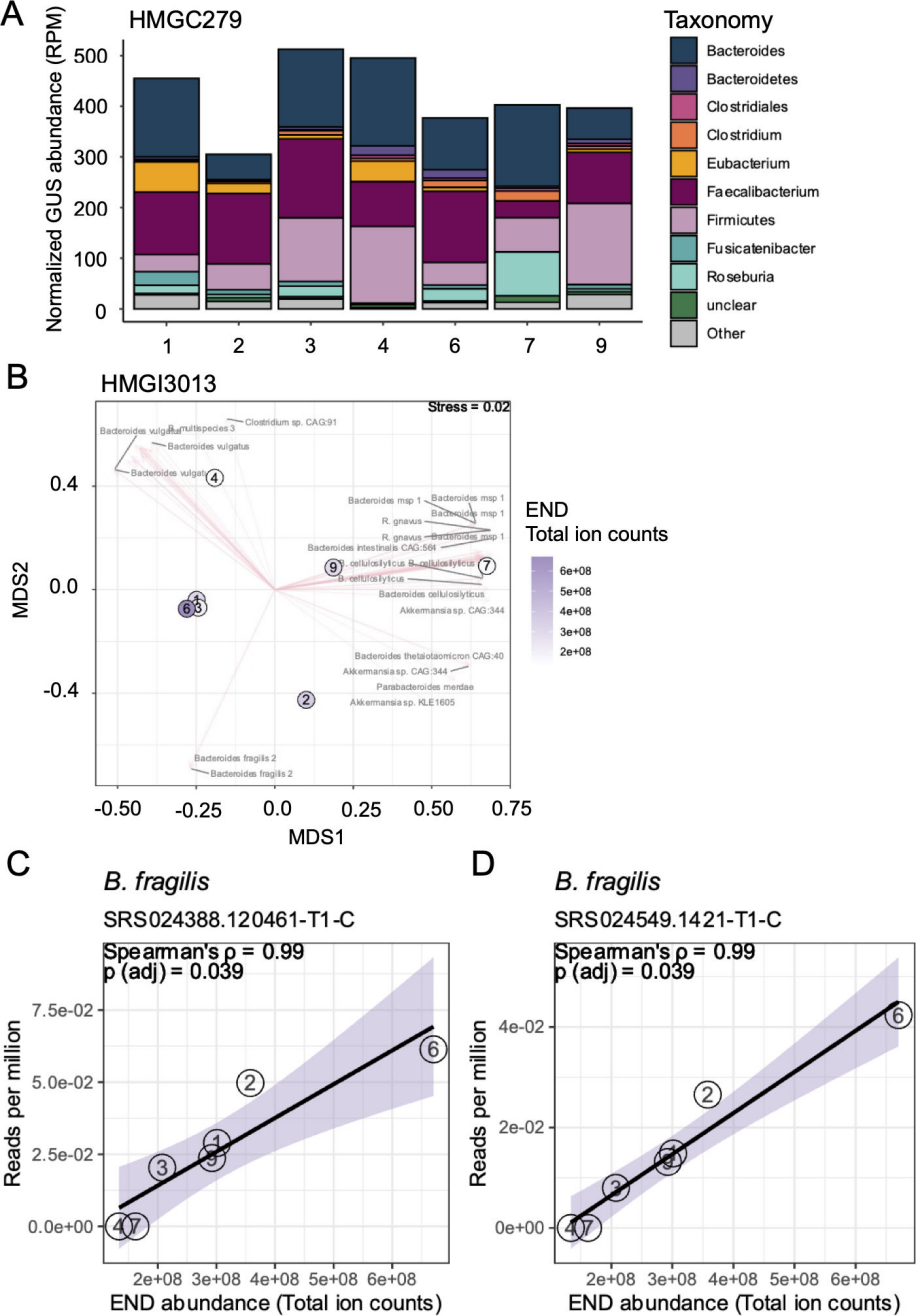
Characterization of whole-genome shotgun data reveals a correlation between END production and *B. fragilis* GUS genes. (**A**) Stacked bar plot showing the normalized abundance, in reads per million (RPM), of bacterial GUS genes (HMGC279) and their assigned taxonomy across multiple fecal donors (1, 2, 3, 4, 6, 7, and 9; samples 5 and 8 were excluded because they could not be matched to their sample IDs). Each color represents a different taxonomic group. (**B**) Non-metric multidimensional scaling (NMDS) plot of HMGI3013 bacterial GUS gene compositions (Bray–Curtis). Each point represents a fecal donor (numbered), and the distances between points reflect the similarities in bacterial GUS gene composition. Points are colored according to END production, and features that separate the points are shown as pink arrows. (C and D) Scatter plots showing the Spearman’s rank correlation (ρ) between END production and normalized HMGI3013 GUS gene abundance, in RPM, per fecal donor (numbered). The black line represents the trend, and the shaded area indicates the 95% confidence interval.

After observing variation in END-G hydrolysis across donors, we sought to examine each donor’s fecal sample and characterize their respective microbial GUS genes to further explain the inter-individual differences in enzyme activity. We performed whole-genome shotgun sequencing (WGS) on the donors’ fecal samples and classified bacterial GUS genes using the curated database produced by Pollet et al. (24). Briefly, these databases were curated using the Human Microbiome Project Stool Sample Catalog data sets. One database, HMGI3013, was generated from 139 individuals in which 3,013 GUS proteins were identified. In a second database, HMGC279, 279 GUS proteins were clustered and consolidated from HMGI3013 to represent only unique GUS gene sequences that share >95% sequence identity. Using the HMGC279 database, we performed an analysis of GUS gene composition within our donor samples. Of GUS gene sequences containing taxonomic assignments, we found slight sample-to-sample variations in the total normalized abundance of GUS genes, as well as the taxon they belonged to (Fig. 7A). Next we performed non-metric multi-dimensional scaling (NMDS) on the same data and observed that donors 1, 3, 4, and 6 clustered together, suggesting that while donor 6 was the strongest hydrolyzer of END, these four donors were compositionally similar in regard to their abundances of GUS gene counts (Fig. S4). Donors 2, 7, and 9 appeared more compositionally dissimilar to others, as indicated by eigenvectors representing characteristic features of each sample. The HMGI3013 annotated data yielded similar but more granular results, with more pronounced clustering in samples from donors 1, 3, and 6 on an NMDS (Fig. 7B).

Next, we performed correlation analysis between GUS gene abundances and each sample’s hydrolysis activity for END-G. Strikingly, in the HMGI3013 data set, hydrolysis of END-G was significantly and positively correlated with two *Bacteroides fragilis* GUS genes (FDR-adjusted *P*-values for each: 0.039; Spearman’s coefficient for each: 0.99) (Fig. 7C and D; Table S9). No significant correlates in the HMGC279 data set were observed for END-G. These two genes shared largely the same sequence identity, differing only by two amino acids. Still, these differences were sufficient to produce differences in the average, unnormalized coverage depth, potentially suggesting that each is a distinct variant of the same *Bacteroides fragilis* GUS gene (Fig. S5).

## DISCUSSION

Tamoxifen stands as a cornerstone in hormone-based therapy for metastatic breast cancer, boasting a 38% reduction in breast cancer irrespective of the age of the woman treated, prolonging both recurrence-free and overall survival rates (33). Within this population, tamoxifen usage affords a 25%–30% decrease in breast cancer mortality (34). Initially categorized as an anti-estrogen due to its inhibition of estrogen receptors in breast tissue, tamoxifen also acts as an agonist for estrogen receptors in specific body areas like the endometrium, liver, and bone (34, 35). This dual role has led to its classification as a selective estrogen-receptor modulator (SERM) (34, 36). Widely employed in the treatment of breast cancer, tamoxifen is also utilized preventatively in women with a high risk of developing the condition (36). Despite its utility, tamoxifen’s efficacy in reducing the risk of breast cancer among women at elevated risk is limited to just 50%, failing to meet the desired level of effectiveness (37). The exact cause for this phenomenon has remained unclear. Despite its potential efficacy, the variable response observed among patients warrants closer examination, particularly concerning the role of the microbiome and the diverse pharmacokinetic profiles across individuals. We have inferred from our collected data and observations that the gut microbiota and its ability to control the drug concentration at therapeutic sites in the body may be crucial for predicting and improving patient responsiveness to tamoxifen. Adding to this, it could be fruitful to determine which specific bacteria interact with the drug and to identify foods that support the growth of these bacteria to provide dietary advice to patients prescribed tamoxifen, especially considering studies that suggest high BMI correlates with reduced systemic endoxifen levels (11).

Metabolism that shapes pharmacokinetics is not isolated to the activity of host enzymes but occurs in conjunction with metabolism by the gut microbiota. Therefore, we tested the hypothesis that variable tamoxifen metabolism results from the interaction of the drug with diverse human gut bacterial communities, which are unique across people (38). Using gnotobiotic- and antibiotics-treated mice, we found that the gut microbiome plays an important role in tamoxifen pharmacokinetics by facilitating tamoxifen absorption or retention/recycling. Notably, both prolonged exposure to the drug and the presence of intestinal bacteria resulted in the greatest abundance of circulating tamoxifen (Fig. 2 and 3), suggesting the adaptation of gut microbial communities to tamoxifen and readiness of these microbes to control the reabsorption of bioactive metabolites into the bloodstream. No detected changes in gut microbiota community composition, overall abundance, nor the cecal metabolome upon tamoxifen exposure (Fig. 4) highlight the importance of examining the gut microbiota at the functional level to determine its impacts on host response. To target the specific metabolism that takes place upon exposure of gut microbes to tamoxifen, we cultured fecal bacteria from healthy human donors with a physiologically relevant dose (60 µM) of tamoxifen (16) (or vehicle alone). The data revealed overall similar metabolomic profiles between five donors in the absence of tamoxifen (Fig. 5; Fig. S3). The addition of tamoxifen, however, resulted in some variability in metabolomic profiles across donors, suggesting that gut microbiotas from different people have differing responses to tamoxifen. This highlighted the inter-individual variation in gut microbiota responses to tamoxifen. For example, donors 4 and 5 showed distinct patterns from other donors, further suggesting that diet and/or other unique lifestyles likely affect gut microbiome compositions and their response to tamoxifen. Separately, we saw an increase in fatty acid abundance following tamoxifen exposure in both aerobic and anaerobic conditions. This suggests altered bacterial lipid metabolism, which may be linked to the fact that tamoxifen is a highly hydrophobic molecule.

In addition, we detected several unknown tamoxifen-derived metabolites (e.g., hydroxylated or potentially glycosylated tamoxifen) using untargeted metabolomics (Tables S7 and S8). This inter-individual difference likely reflects bacteria species-specific handling of tamoxifen, such as degradation or bioaccumulation (14, 39). Identifying bacteria-mediated degradation products of tamoxifen remains an ongoing consideration in elucidating the variable efficacy of tamoxifen for patients with recurrent breast cancer. Identification and quantitation of such metabolic products of tamoxifen will give hints for the identification of the responsible bacterial species and their metabolic pathways. These signals may also be used as fecal biomarkers for the prediction of tamoxifen bioavailability in patients. Altogether, the metabolomic data highlight the importance of functional assessment of gut microbes.

Because glucuronidation is a primary metabolic pathway for the host metabolism of tamoxifen and because hydrolyzing these glucuronides is needed for the drug to reach therapeutic concentrations in the bloodstream, we investigated the role of bacterial β-glucuronidases in this process. To this end, we incubated 4HT-G and END-G with fecal bacteria from healthy human donors and followed field-standard methods for evaluating β-glucuronidase activity (40–42) (Fig. 6). The data revealed intriguing insights into drug metabolism and its implications for explicit variations among individuals. Through incubation of cell lysates from fecal samples from nine different donors with each respective substrate, it became evident that there exists a notable diversity in the levels of glucuronide hydrolysis across both individual donors and between the two metabolites tested. Notably, 4HT-G was consistently and efficiently hydrolyzed by glucuronidase enzymes across all donors, implying a uniform activity in this specific pathway (Fig. 6D). However, the hydrolysis of END-G exhibited significant variability, with donor 6 displaying the highest efficacy of hydrolysis (Fig. 6C). Toward identifying the bacteria responsible for this effect, we found that END-G hydrolysis is significantly and positively correlated with the abundance of two GUS-encoding genes in *Bacteroides fragilis* (Fig. 7C and D; Table S9), an organism that expresses structurally and functionally unique types of GUS enzymes (24, 43). These findings highlight the critical role of inter-individual variations in β-glucuronidase levels, strains, and possibly types of β-glucuronidases. Such variations across peoples’ gut microbiomes may lead to divergent levels of exposure to bioactivated metabolites among patients undergoing tamoxifen treatment. Consequently, these differences in drug metabolism could potentially influence the therapeutic efficacy and adverse effects experienced by patients.

By elucidating the interplay between gut bacterial β-glucuronidase activity and drug metabolism, this study contributes to a deeper understanding of pharmacogenomics that is needed to achieve individualized and more effective therapeutic interventions in clinical practice. Furthermore, this study elucidates the critical role of the gut microbiome and the importance of accounting for this collection of microbes when studying the metabolism and pharmacokinetics of medications. Advancing understanding of these interactions will enhance the effectiveness of therapeutic strategies and ultimately improve patient outcomes in breast cancer treatment.

## MATERIALS AND METHODS

### Human fecal sample collection

Human fecal samples were collected from nine healthy human participants using GutAlive (MicroViable Therapeutics) to collect and preserve the anaerobic nature of the microbial communities. All participants consented to take part in the research, and the study received approval from the Institutional Review Board at the University of California, Irvine. Samples were transferred to the laboratory within a few hours of collection and immediately moved to the anaerobic chamber for aliquoting. Fecal slurries were resuspended in pre‐reduced phosphate buffer saline (PBS; pH 7.4) at a final concentration of 0.1 g mg/mL. The suspensions were vortexed for 5 minutes and then allowed to settle for another 5 minutes before being frozen in a −80°C freezer until use in further experiments.

### Mouse experiments

#### Animals

Animal experiments were conducted following approval by the Institutional Animal Care and Use Committee at the University of California, Irvine. Germ-free, wild-type mice (BALB/c; originally from the University of North Carolina National Gnotobiotic Rodent Resource Center) were humanized using a cocktail of human fecal samples from five healthy human donors. Donor samples were chosen for inclusion in the study according to the Bristol stool scale (only Types 3–5 were included). Gnotobiotic mice were housed and bred in sterile gloveboxes (isolators) fitted with HEPA filters (Class Biologically Clean, Madison, WI) inside UC Irvine’s AAALAC-accredited animal-care facility. Both male and female mice were bred, though only female mice at a mature age (3 months of age) were used for experiments, as liver function differs between juvenile and adult mice and liver function is a key variable in the metabolism of tamoxifen (44). Also, breast cancer and tamoxifen treatment are more relevant in the female context. To minimize data clustering due to cage effects, mice were housed in two per cage. Mice were fed food and water *ad libitum* and housed on a 12-hour light/dark cycle. Animal-care supervision and evaluation of mice occurred daily.

#### Fecal microbiota transplantation and tamoxifen administration

In most murine research settings, fecal microbiota transplantation (FMT) is predominantly conducted through oral gavage. Gnotobiotic mice were either maintained germ-free or they were colonized with bacterial communities from the fecal samples of healthy human donors. Oral gavage was used in our experiments to colonize mice with bacteria and to introduce tamoxifen (both unlabeled and ^13^C labeled). Control mice were dosed with respective treatment vehicles so that all mice were handled in identical ways. Mice were randomized into treatment and control groups, to the extent possible.

#### Antibiotic treatment

The following cocktail of antibiotics were prepared to deplete the gut microbiome in wild-type C57BL/6J mice: 0.3 g ampicillin, 0.3 g neomycin, 0.3 g metronidazole, and 0.15 g vancomycin into 0.6 L of water. Aspartame (1.5% [wt/vol]) was added to enhance the drinking preferences of the mice.

#### Isotope tracing

To trace metabolites through the host organs and intestinal microbiome, mice were orally gavaged with a 1:1 ratio of ^13^C-labeled:unlabeled tamoxifen. Animals were monitored for signs of distress (including but not limited to labored breathing, difficulty ambulating, poor feeding, or splinting) and no signs of distress were observed.

#### Tissue harvest

Blood and tissues were harvested as previously described (45). Blood was collected via tail snip for non-terminal pharmacokinetic tracer analysis into microcentrifuge tubes (or capillary blood collection tubes) and placed on ice for 20 minutes to allow clotting for serum collection. Blood collection tubes were spun at 16,000 rcf for 10 minutes at 4°C and supernatant (serum) was collected and stored at −80°C until LC–MS. Tissues were placed between a folded-in-half heavy aluminum foil sheet, smashed with a precooled Wollenberger clamp, and frozen in liquid nitrogen.

### Tamoxifen/metabolite extraction method from *in vitro* incubations

Human fecal samples were incubated with or without tamoxifen for 72 hours under both aerobic and anaerobic conditions in Gifu anaerobic medium (GAM), which is thought to reasonably mimic the nutrient environment of the gut because culturing fecal samples in this medium maintains the fecal samples’ bacterial community structure and optimizes taxonomic diversity (46). This time point was chosen because gut transit times up to 72 hours are considered normal (47). Ethyl acetate (1 mL) was used as an extraction solution for 200 µL of culture. Samples were vortexed for 15 seconds on max speed and centrifuged at 10,000 rcf for 1 min to cleanly separate layers. The top organic layer (750 µL) was collected and transferred to a new microcentrifuge tube. Nitrogen gas was used to dry down the sample before being resuspended in 750 µL of 1:1 (vol/vol) acetonitrile:water. The supernatant was then transferred into an ice-cold, pre-labeled mass spectrometer vial (Thermo Scientific, Cat# 200046) for LC–MS injection.

### Tamoxifen/metabolite extraction from mouse tissues and serum

Ice-cold acetonitrile/methanol/water (40:40:20) extraction solution was added to the tissue powder to make 25 mg tissue per 1 mL of solution. This mixture was vortexed for 10 s and then placed on ice. For serum, 5 µL of serum was diluted to 150 µL with ice-cold acetonitrile/methanol/water extraction mixture, vortexed for 10 s, and placed on ice. Samples were centrifuged at 16,000 rcf for 10 min at 4℃ and minimal amounts of supernatant were collected (to avoid touching pellets): ~100 µL for tissue extraction and ~50 µL for serum extraction were sufficient. The supernatant was then transferred into an ice-cold, pre-labeled mass spectrometer vial (Thermo Scientific, Cat# 200046) for LC–MS injection.

### LC–MS

A quadrupole orbitrap mass spectrometer (Q Exactive Plus; Thermo Fisher Scientific) operating in negative and positive modes was coupled to a Vanquish UHPLC system (Thermo Fisher Scientific) with electrospray ionization and used to scan from *m*/*z* 70 to 1,000 at 2 Hz, with a 140,000 resolution. LC separation was achieved on an XBridge BEH Amide column (2.1  ×  150  mm^2^, 2.5 µm particle size, 130 Å pore size; Waters Corporation) using a gradient of solvent A (95:5 water:acetonitrile with 20 mM of ammonium acetate and 20 mM of ammonium hydroxide, pH 9.45) and solvent B (acetonitrile). The flow rate was 150  µL min^−1^. The LC gradient was 0 min, 90% B; 2 min, 90% B; 3 min, 75% B; 5 min, 75% B; 7 min, 75% B; 8 min, 70% B; 9 min, 70% B; 10 min, 50% B; 12 min, 50% B; 13 min, 25% B; 14 min, 20% B; 15 min, 20% B; 16 min, 0% B; 20.5 min, 0% B; 21 min, 90% B; and 25 min, 90% B. The autosampler temperature was 4°C and the injection volume was 3  µL. Data were analyzed using MAVEN software (build 682, http://maven.princeton.edu/index.php). Natural isotope correction for dual isotopes was performed with AccuCor2 R code (https://github.com/wangyujue23/AccuCor2) and IsoCorrectoR (48). Serum and tissue levels of tamoxifen and 4HT were calculated using a standard curve for each metabolite generated by LC–MS. In Fig. 3, one outlier mouse from the TAM group was removed by an outlier test (49).

### 16S rRNA quantitative PCR

To assess bacterial load following antibiotics treatment of the humanized mice, bacterial DNA was extracted from fecal pellets (10–20 mg) using Quick-DNA Fecal/Soil Microbe Kits (Zymo Research) according to the manufacturer’s instructions. Purified DNA was amplified by qPCR using SYBR green master mix (Life Technologies) on QuantStudio6 Real-Time PCR system (Life Technologies). DNA from the *E. coli* DH5a strain was used as a standard for determining the copy number of the 16S rRNA bacterial gene by qPCR. Primer pairs for the bacterial universal 16S rRNA gene were as follows: forward, GTGGTGCACGGCTGTCGTCA; reverse, ACGTCATCCACACCTTCCTC. Relative bacterial abundances were calculated from Ct values relative to universal bacterial abundance in each sample.

### Extraction of β-glucuronidase from human fecal samples

Human fecal samples were collected from nine healthy people using GutAlive (MicroViable Therapeutics) to collect fecal samples in anaerobic conditions. All participants consented to take part in the research, and the study received approval from the Institutional Review Board at the University of California, Irvine. The fecal samples were transferred from a −80°C freezer to a biosafety cabinet (BSC) and thawed on ice. Roche Complete protease inhibitor cocktail (1 tablet per 50 mL) was added to 50 mL of extraction buffer (25 mM HEPES buffer, pH 7.4, with 25 mM NaCl) and incubated for 15 minutes at room temperature. Approximately 100 mL of autoclaved garnet beads (7 mm, OMNI) were added to 15 mL conical tubes (Corning). From each of the nine donors, 0.5 g of human fecal samples was collected and transferred, individually, to 15 mL tubes and then resuspended in 2.5 mL of the extraction buffer and vortexed vigorously for 10 seconds. Next, samples were centrifuged at 300 × *g* for 5 minutes at 4°C. The resulting fecal supernatant was decanted into a new 15 mL conical tube, and the remaining pellet was resuspended in 2.5 mL of fresh extraction buffer. The vortexing and centrifugation steps mentioned above were repeated, and the resulting fecal supernatant, free of fibers, was combined with the supernatant of the first round and centrifuged at 300 × *g* for 5 minutes at 4°C. To lyse the cells, the fecal supernatant was divided into 1.5 mL amounts in lysing matrix tubes (MPbio) and transferred to the Mini-Beadbeater-96 (BioSpec Products, 115 V). Bead-beating was performed for 30 seconds, and the procedure was repeated twice with a minute of cooling the samples in a 4°C refrigerator in between repetitions. After lysis, the samples were centrifuged at 18,000 × *g* for 30 minutes at 4°C. The supernatant was concentrated and buffer-exchanged using 30 kDa MWCO AmiconUltra concentrators to remove metabolites. Bradford Assay was performed to determine total protein concentration utilizing a nanodrop (Thermo Scientific).

### Sequence library preparation

Samples were harvested at the terminal endpoint of the gnotobiotic experiment and processed and analyzed with the Zymo Research Microbiome Sequencing Service (Zymo Research, Irvine, CA). DNA from cecal samples of the humanized + TAM and the humanized + vehicle mouse groups were extracted using the ZymoBIOMICS−96 MagBead DNA Kit (Zymo Research, Irvine, CA). The ZymoBIOMICS Microbial Community Standard (Zymo Research, Irvine, CA) was used as a positive control for each DNA extraction. Bacterial 16S ribosomal RNA gene-targeted sequencing was performed using the *Quick*-16S NGS Library Prep Kit (Zymo Research, Irvine, CA). The V3–V4 region of the 16S rRNA gene was amplified, and the primers used were as follows: V3V4_341f_p1_n6: NCCTACGGGDGGCWGCAG, V3V4_341f_p2_n4: NCCTAYGGGGCGCWGCAG, V3V4_341f_p3_n1: NCCTACGGGGTGCAGCAG, V3V4_341f_p4_n1: GCCTACGGGAGGCTGCAG, V3V4_806r_p1_n24: NGACTACNVGGGTMTCTAATCC, V3V4_806r_p2_n4: NGACTACNAGGGTATCTAATCC, V3V4_806r_p3_n3: NGACTACDCAGGTCTCTAATCT, V3V4_806r_p4_n2: NGAMTACCGGGGTTTCTAATCC, V3V4_806r_p5_n1: NGACTACCAGGGTATCTAAGCC. The final PCR products were quantified with qPCR fluorescence readings and pooled together based on equal molarity. The final pooled library was cleaned with the Select-a-Size DNA Clean & Concentrator (Zymo Research, Irvine, CA), then quantified with TapeStation (Agilent Technologies, Santa Clara, CA) and Qubit (Thermo Fisher Scientific, Waltham, WA). The ZymoBIOMICS Microbial Community DNA Standard (Zymo Research, Irvine, CA) was used as a positive control for each targeted library preparation. Negative controls (i.e., blank extraction control and blank library preparation control) were included to assess the level of bioburden carried by the wet-lab process. The final libraries were sequenced using Illumina MiSeq with a v3 reagent kit (600 cycles). The sequencing was performed with a 10% PhiX spike-in.

Unique amplicon sequence variants were inferred from raw reads using the DADA2 pipeline (50). Potential sequencing errors and chimeric sequences were also removed with the DADA2 pipeline. Taxonomy assignment was performed using Uclust from Qiime v.1.9.1 with the Zymo Research Database, a 16S database that is internally designed and curated, as reference. Composition visualization, alpha-diversity, and beta-diversity analyses were performed with Qiime v.1.9.1 (51). If applicable, a taxonomy that has significant abundance among different groups was identified by LEfSe (52) using default settings. Other analyses such as heatmaps, Taxa2ASV Decomposer, and PCoA plots were performed with internal scripts.

### Correlation analysis between microbial composition and ^13^C-tamoxifen abundance

Spearman rank-order correlation coefficients were calculated to assess the relationship between ^13^C-tamoxifen abundance (both AUC and 2 hour only) and the relative abundance of each microbial taxon within the cecal contents of both the humanized + TAM and the humanized + vehicle mouse groups. For each taxon, a *P*-value was calculated to assess the significance of the observed correlation. Corrections for multiple comparisons were applied to control the false-discovery rate (FDR) and minimize the likelihood of errors. The Benjamini–Hochberg procedure was applied to control the AUC FDR (Table S5) and the 2-hour FDR (Table S6) at a significance level of *P* < 0.05.

### Detection of glucuronide-free metabolites

Fecal lysates (0.1 mg/mL from each of nine human donors) were individually incubated with 0.8 mM of *N*-desmethyl-4-hydroxytamoxifen-β-D-glucuronide (*E*/*Z* mixture; END-G) and (*E*)-4-hydroxy tamoxifen *O*-β-D-glucuronide (4HT-G), individually, in 50 mM HEPES and 25 mM NaCl (pH 6.5) for 180 minutes at 37°C with shaking at 300 rpm. Concurrently, samples containing 0.8 mM (*Z*)-4-hydroxytamoxifen in 50 mM HEPES and 25 mM NaCl (pH 6.5) were prepared as controls. All samples were incubated in anaerobic conditions. To conduct the time-course analysis, six replicate samples were prepared for each donor; one of these samples was quenched every 30 minutes from 0 to 180 minutes. All reactions were quenched with 0.6 M sodium carbonate. Samples were flash-frozen in an ethanol bath and then stored at −80°C until analysis by LC–MS.

### Whole-genome shotgun sequencing and metagenomic analysis

Shotgun sequencing data were produced from the nine samples of human fecal donor DNA. An average of 51,277,301 ± 23,647,916 (σ) raw reads per sample were obtained. Raw reads were processed using BBMap v38.96 to remove sequencing artifacts, eukaryotic DNA, and poor-quality sequences, resulting in an average of 45,963,998 ± 21,066,480 (σ) quality filtered reads per sample. Two of the nine samples were excluded due to not being able to match the samples to their IDs. To classify bacterial GUS genes, we used the curated database produced by Pollet et al. (24). Briefly, quality-filtered sequences were aligned against both HMGI3013 and HMGC279 databases produced by Pollet et al., which contained total GUS gene sequences that were detected by the Human Microbiome Project versus only unique GUS gene sequences (which were derived from HMGI3013), respectively. This was performed using DIAMOND v2.0.15 using the ultra-sensitive preset and a minimum sequence identity of 90%. For reads that mapped to multiple GUS genes equally, the read count was divided by the total number of equivalent features. This produced a mean of 19,251 ± 8,941 (σ) reads mapping to the HMGC279 database and a mean of 19,807 ± 9,184 (σ) reads mapping to the HMGI3013 database per sample. GUS-derived reads were normalized to reads per million (RPM) before analysis in R. Non-metric multidimensional scaling (NMDS) was performed using the “metaMDS” function from the Vegan package, with the distance metric set to “Bray–Curtis.” The “envfit” function within the Vegan package was used to display loading vectors within the NMDS plot. Spearman’s rank correlations were performed to correlate either *N*-desmethyl-4-hydroxytamoxifen β-D-glucuronide or (*E*)-4-hydroxy tamoxifen *O*-β-D-glucuronide hydrolysis with normalized GUS abundance using the “rcorr” function from the Hmisc package. *P*-values obtained from the “rcorr” function were adjusted for multiple comparisons using the Benjamini–Hochberg method. Taxonomies for the SRS024549.1421-T1-C and SRS024388.120461-T1-C amino acid sequences were identified using NCBI’s blastp with default parameters against the latest version of the non-redundant (nr) database. All data were visualized using “ggplot2” in R. The code used for analysis is available: https://github.com/Javelarb/Bess_lab_tamoxifen.

## Data Availability

All data needed to evaluate the conclusions in the paper are present in the paper or the supplemental materials and/or associated with BioProject Accession number PRJNA1176874.

## AFTER EPUB

*[This article was published on 25 November 2024 with errors in Acknowledgments. The Acknowledgments were corrected in the current version, posted on 11 December 2024.]*

## SUPPLEMENTAL MATERIAL

Supplemental material mbio.01679-24-s0001.docx Supplemental figures and captions for supplemental tables.

Table S1 mbio.01679-24-s0002.xlsx Taxonomic classification of microbial composition at the species level related to Fig. 4A.

Table S2 mbio.01679-24-s0003.xlsx Taxonomic classification of microbial composition at the phylum level related to Fig. S2A.

Table S3 mbio.01679-24-s0004.xlsx Taxonomic classification of microbial composition at the genus level related to Fig. S2B.

Table S4 mbio.01679-24-s0005.xlsx Metabolomic analysis of cecal content harvested from humanized mice treated with tamoxifen or vehicle related to Fig. 4E.

Table S5 mbio.01679-24-s0006.xlsx Correlation analysis between microbial composition and ^13^C-tamoxifen abundance after FDR correction.

Table S6 mbio.01679-24-s0007.xlsx Correlation analysis between microbial composition and ^13^C-tamoxifen abundance after FDR correction at 2 hours post-tamoxifen administration.

Table S7 mbio.01679-24-s0008.xlsx Metabolomic analysis of tamoxifen-treated human gut bacterial cultures under anaerobic conditions related to Fig. 5.

Table S8 mbio.01679-24-s0009.xlsx Metabolomic analysis of tamoxifen-treated human gut bacterial cultures under aerobic conditions related to Fig. S3.

Table S9 mbio.01679-24-s0010.xlsx Correlation analysis between GUS gene abundances and END-G or 4HT-G hydrolysis activity by human fecal bacteria.

ASM does not own the copyrights to Supplemental Material that may be linked to, or accessed through, an article. The authors have granted ASM a non-exclusive, world-wide license to publish the Supplemental Material files. Please contact the corresponding author directly for reuse.
