# Supplementary material for: Variation in human gut microbiota impacts tamoxifen pharmacokinetics
Source: mBio. 2024 Nov 25;16(1):e01679-24. doi: 10.1128/mbio.01679-24 (PMC11708054; doi:10.1128/mbio.01679-24)
Supplement: Supplemental material — Supplemental figures and captions for supplemental tables. [file mbio.01679-24-s0001.docx]

**Supplementary Figures (see Supplementary Table Legends at end of file)**


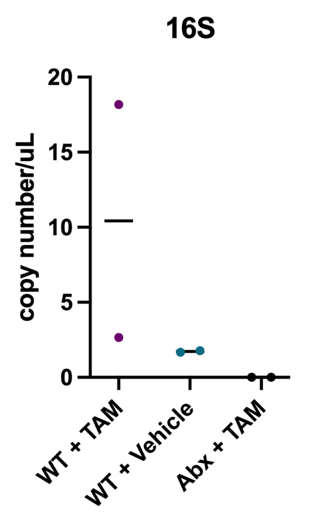


**Supplemental Figure 1. Bacterial load in fecal samples of antibiotics-treated mice exposed to tamoxifen or a vehicle control.** qPCR quantification of 16S rRNA gene copies as a function of antibiotic treatment and following treatment with tamoxifen or vehicle for 10 days.


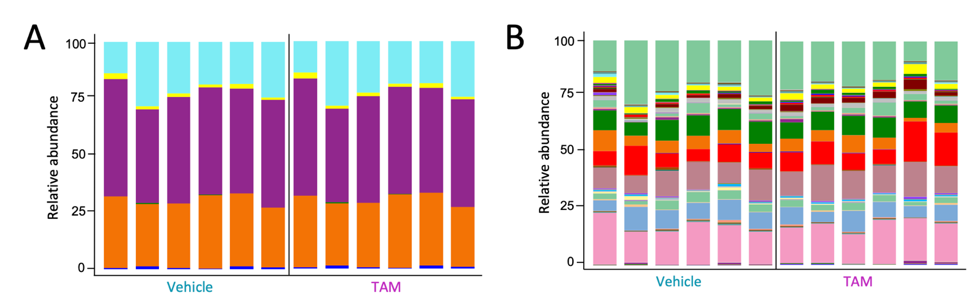


**Supplemental Figure 2. Diversity and abundance of bacteria in the gut microbiome of tamoxifen-treated mice at phylum and genus levels.** (A) Taxonomic analysis of microbial composition at the (A) phylum and (B) genus level from cecal samples of the humanized + vehicle and the humanized + TAM mouse groups. The size of the bars represents the relative abundance of the bacterial taxa. Different taxa were shaded by different colors; see raw data (Table S2 and S3) for legend.


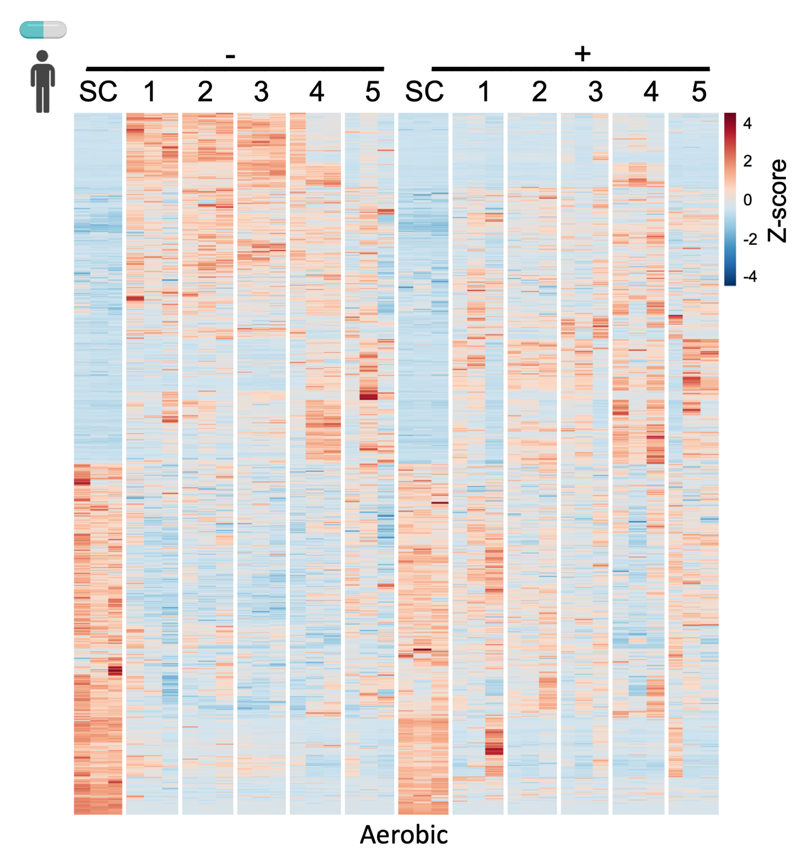


**Supplemental Figure 3. Metabolomic analysis of tamoxifen-treated human gut bacterial cultures under aerobic conditions.** (A) Heatmap showing relative abundances of known and unknown metabolites in *ex vivo* cultures of fecal samples incubated with tamoxifen (right) or vehicle control (left) under aerobic conditions. Rows indicate metabolites (Table S8) and columns represent each donor sample. N=5 fecal samples (one per donor) and 3 biological replicates per sample. SC, sterile control (without bacteria).


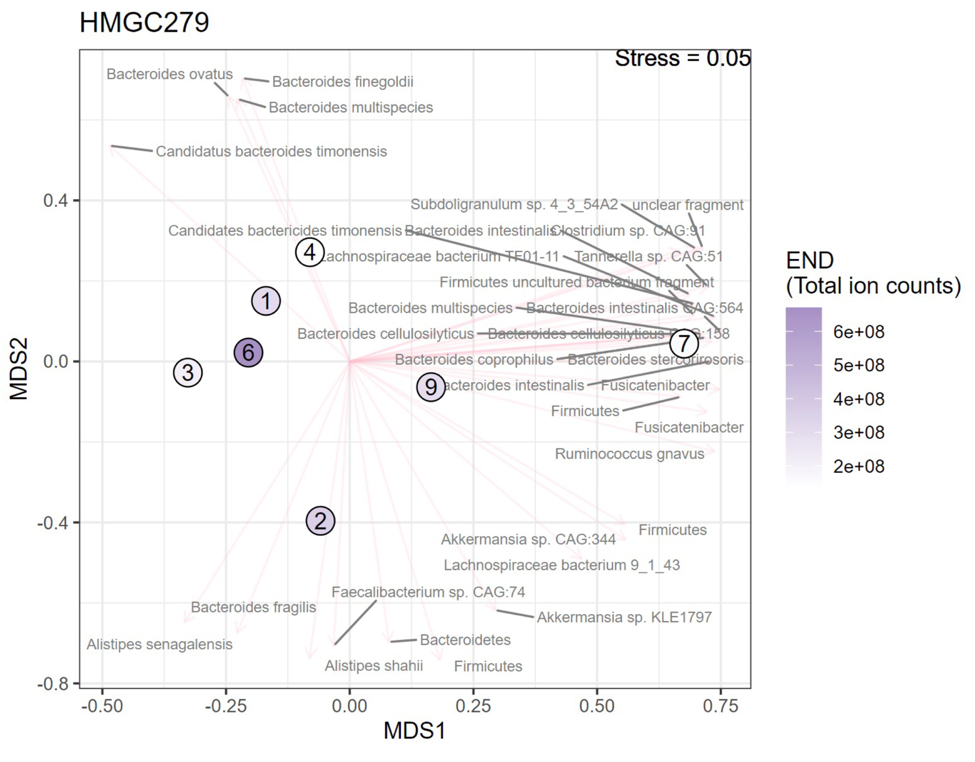


**Supplemental Figure 4**. **Non-metric multidimensional scaling (NMDS) plot of HMGC279 bacterial GUS gene compositions (Bray-Curtis).** Each point represents a fecal donor (numbered), and the distances between points reflect the similarities in bacterial GUS gene composition. Points are colored according to END total ion counts, and taxonomic features that separate the points are shown as pink arrows.


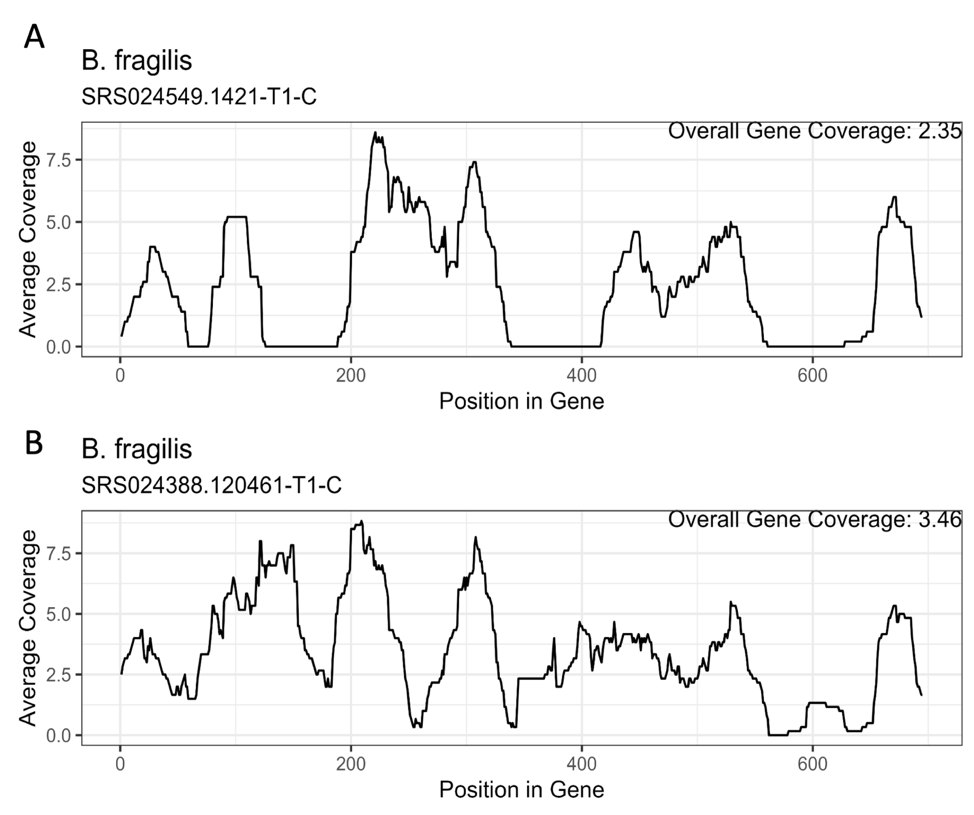


**Supplemental Figure 5. Overall gene coverage across two different *Bacteroides fragilis* GUS genes that correlate with hydrolysis of tamoxifen glucuronides.** (A & B) Plots displaying the average, unnormalized coverage depth of two *Bacteroides fragilis* GUS genes found to be significantly correlated with N-Desmethyl-4-hydroxytamoxifen b-D-glucuronide hydrolysis among seven fecal donors. The x-axis shows the position within the gene, and the y-axis represents the average sequencing coverage. The overall gene coverage refers to the average sequencing coverage throughout the entire length of the gene.

**Supplemental Table Legends**

**Table S1. Taxonomic classification of microbial composition at the species level related to Figure 4A.** Due to the absence of traditional biological classification systems, Operational Taxonomic Unit (OTU) IDs define the microbial individuals at different taxonomic levels. The table indicates the percentages of each OTU across samples. Tamoxifen-treated mice showed no significant difference in abundances at the species level as compared to vehicle-treated mice. N=6 mice. Abbreviations: k: kingdom; p: phylum; c: class; o: order ; f: family ; g: genus ; s: species.

**Table S2. Taxonomic classification of microbial composition at the phylum level related to Figure S2A.** Due to the absence of traditional biological classification systems, Operational Taxonomic Unit (OTU) IDs defines the microbial individuals at different taxonomic levels. The table indicates the percentages of each OTU across samples. Tamoxifen-treated mice showed no difference in abundances at the phylum level compared to vehicle. N=6 mice. Abbreviations: k: kingdom; p: phylum.

**Table S3. Taxonomic classification of microbial composition at the genus level related to Figure S2B.** Due to the absence of traditional biological classification systems, Operational Taxonomic Unit (OTU) IDs defines the microbial individuals at different taxonomic levels. The table indicates the percentages of each OTU across samples. Tamoxifen-treated mice showed no difference in abundances at the genus level compared to vehicle. N=6 mice. Abbreviations: k: kingdom; p: phylum; c: class; o: order ; f: family ; g: genus.

**Table S4. Metabolomic analysis of cecal content harvested from humanized mice treated with tamoxifen or vehicle related to Figure 4E.** Relative abundances of the top 25 known and unknown metabolites found in the cecal content of mice treated with tamoxifen or vehicle control for 10 days. N=6 mice/group.

**Table S5. Correlation analysis between microbial composition and 13C-tamoxifen abundance after FDR correction.** Abundance of bacterial taxa was correlated with circulating levels of 13C-tamoxifen (as measured by area under the curve in Figure 2C). No significant correlation between bacterial taxa and 13C-tamoxifen AUC was detected at a significance threshold of *P*<0.05.

**Table S6. Correlation analysis between microbial composition and 13C-tamoxifen abundance after FDR correction at 2-hours post-tamoxifen administration.** Abundance of bacterial taxa was correlated with circulating levels of 13C-tamoxifen (as measured by area under the curve at 2-hours post-administration of tamoxifen in Figure 2C). Two significant correlates were identified: Clostridia (Firmicutes; Spearman Correlation = 0.855, FDR-corrected *P*-value = 0.0466 and Erysipelotrichia (Firmicutes; Spearman Correlation = 0.854, FDR-corrected *P*-value = 0.0466).

**Table S7.** **Metabolomic analysis of tamoxifen-treated human gut bacterial cultures under anaerobic conditions related to Figure 5.** Relative abundances of known and unknown metabolites in *ex vivo* cultures of fecal samples incubated with tamoxifen or vehicle control under anaerobic conditions. N=5 fecal samples (one per donor) and 3 biological replicates per sample.

**Table S8.** **Metabolomic analysis of tamoxifen-treated human gut bacterial cultures under aerobic conditions related to Figure S3.** Relative abundances of known and unknown metabolites in *ex vivo* cultures of fecal samples incubated with tamoxifen or vehicle control under aerobic conditions. N=5 fecal samples (one per donor) and 3 biological replicates per sample.

**Table S9. Correlation analysis between GUS gene abundances and END-G or 4HT-G hydrolysis activity by human fecal bacteria.** Spearman's rank correlations were performed to correlate either END-G or 4HT-G hydrolysis with normalized GUS gene abundance. Spearman correlation coefficients (Rho) and corresponding *p*-values measure the strength and significance of associations between GUS gene abundance and hydrolysis activity by human fecal bacteria. *P*-values were adjusted for multiple comparisons using the Benjamini-Hochberg method. Gene IDs and NCBI identifiers correspond to specific genes that were found in each biological sample.
